# *QuickStats*: Percentage* of Children and Adolescents Aged 0–17 Years Who Have Experienced a Specified Stressful Life Event,^†^ by Type of Event and Poverty Status^§^ — National Health Interview Survey, United States, 2019^¶^

**DOI:** 10.15585/mmwr.mm7034a7

**Published:** 2021-08-27

**Figure:**
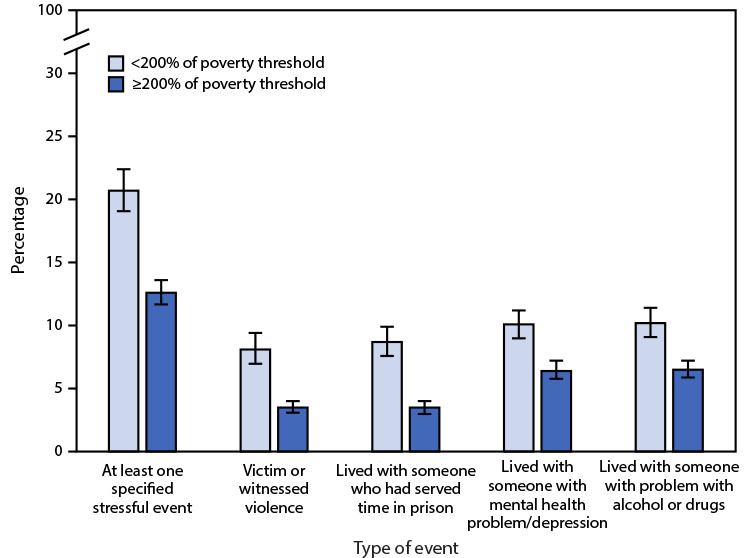
In 2019, 20.7% of children and adolescents in families with incomes <200% of the poverty threshold and 12.6% of children and adolescents in families with incomes ≥200% of the poverty threshold had experienced at least one specified stressful life event. Children and adolescents in families with incomes <200% of the poverty threshold were more likely than children and adolescents in families with incomes ≥200% of the poverty threshold to have been the victim or witnessed violence (8.1% versus 3.5%); lived with someone who had been in jail (8.7% versus 3.5%); lived with a person with problems with mental health or depression (10.1% versus 6.4%); or lived with a person with problems with alcohol or drugs (10.2% versus 6.5%).

**Source:** National Health Interview Survey, 2019. https://www.cdc.gov/nchs/nhis.htm

For more information on this topic, CDC recommends the following link: https://www.cdc.gov/injury/priority/aces.html

